# Improved final predicted height with the injection of leuprolide in children with earlier puberty: A retrospective cohort study

**DOI:** 10.1371/journal.pone.0185080

**Published:** 2017-10-03

**Authors:** Yi-Chun Lin, Chih-Ying Lin, Siew-Yin Chee, Hung-Rong Yen, Fuu-Jen Tsai, Chiu-Ying Chen, Chung-Hsing Wang

**Affiliations:** 1 Department of Chinese Medicine, China Medical University Hospital, Taichung City, Taiwan; 2 Department of Public Health, Doctoral Program, China Medical University, Taichung, Taiwan; 3 Department of Genetics and Metabolism, Children’s Hospital of China Medical University, Taichung, Taiwan; 4 Research Center for Traditional Chinese Medicine, Department of Medical Research, China Medical University Hospital, Taichung, Taiwan; 5 School of Chinese Medicine, College of Chinese Medicine, China Medical University, Taichung, Taiwan; 6 Department of Biotechnology, Asia University, Taichung, Taiwan; 7 Department of Public Health, China Medical University, Taichung, Taiwan; Universite de Rouen, FRANCE

## Abstract

The adult height of children with early onset puberty is limited by the premature maturation of hypothalamic-pituitary-gonadal axis. To evaluate the effects of gonadotropin-releasing hormone analog (GnRHa) treatment on the final height (FH) and bone maturation rate (BMR) in girls with early puberty (EP) or idiopathic central precocious puberty (ICPP), we examined data from girls who were diagnosed with EP or ICPP and underwent GnRHa (Leuplin Depot: 3.75 mg/month) at China Medical University Hospital, in Taiwan, between 2006 and 2015. Patients were observed until the achievement of FH and divided into an “EP group” (T-ep) and “ICPP group” (T-icpp) according to the age of onset of puberty. Eighty-seven patients were enrolled (T-ep, N = 44, puberty onset at 8–10 years; T-icpp, N = 43, puberty onset before 8 years). The demographic data of girls with EP or IPP was characterized. BMR, change in predicted final height (PFH) after GnRHa treatment, target height (TH) and FH were measured. After GnRHa treatment, the study groups (T-ep: 160.24±6.18 cm, T-icpp: 158.99±5.92 cm) both had higher PFH than at initiation (T-ep: 159.83±7.19 cm, T-icpp: 158.58±5.93 cm). There was deceleration of BMR in both groups (T-ep: 0.57±0.39; T-icpp: 0.97±0.97) and a significant difference between the groups (p = 0.027). The gap in FH standard deviation scores (SDS) and TH SDS had a significant difference in T-ep (p = 0.045) but not in T-icpp. Moreover, there was no difference in the gap of PFH SDS between the 1^st^ and final treatment in both groups. We concluded that GnRHa decelerated BMR in girls with earlier puberty. Further prospective clinical studies are warranted.

## Introduction

Puberty marks the end of childhood and is a period when individuals undergo physiological and psychological changes to achieve sexual maturation and fertility [1]. Earlier puberty includes precocious puberty (PP) and early puberty (EP); PP is defined by the appearance of the secondary sexual characteristics before the age of 8 in girls and 9 in boys, whereas EP defined by the appearance of the secondary sexual characteristics between the age of 8 and 10 in girls and 9 and 11 in boys. Several studies have shown that the early onset of puberty may lead to a shorter adult height due to early skeletal maturation and early closure of the epiphyses [2], as well as increase the incidence of cardiovascular disease and diabetes [3, 4]. Furthermore, EP is a complex condition like PP, combining somatic symptoms with negative sequelae, including an increased risk of depression [5], behavioral disorders [6], distorted body perception[7], and hyperactivity/inattention in girls[8].

PP is classified as central or peripheral PP according to the primary source of hormonal production [9]. When PP results from premature maturation of the hypothalamic-pituitary-gonadal axis, the condition is called central PP (CPP). It is important to emphasize the early detection of pathologic PP, such as central nervous system abnormalities, brain tumors, adrenal pathologic problems, gonadal tumors, and genetic problems through a comprehensive history and physical examination [9–11]. Approximately 10–20% of girls [12–14] and the majority of boys have an underlying pathologic cause, whereas the remaining children have an idiopathic etiology, categorized as idiopathic PP (IPP) [15]. Without treatment, discrepancies between physical and chronological age in children with PP may reduce the adult height. The use of gonadotropin-releasing hormone analogues (GnRHa) has become the first-line therapy for CPP [11, 16]. Several studies have shown that GnRHa slows pubertal progression and bone maturation and improves adult stature [17, 18].

When considering intervention for CPP, it is important to distinguish patients according to age. Many patients are referred to pediatric endocrinology clinics for EP [19]. EP is defined as puberty that begins at an earlier age than the age accepted as “normal”. There is no single age range which defines EP; in girls, studies have reported age ranges of 8–10 years [20], 7.5–8.5 years [21], and 8–9 years [22]. EP is a paraphysiological condition, in reference to the earlier appearance of pubertal signs [23]. Such children can reach Tanner stage 2 between 8 and 10 years of age or menarche between 10 and 12 years of age, rather than the mean age of 10.8 (10.7–10.9) years for thelarche and 12.4 (12.2–12.5) years for menarche [24]. This condition may lead to psychosocial problems and stunted growth. However, to date, there are few therapeutic management options and data on the effectiveness of treatment in this group [17, 21].

Therefore, we took a pragmatic approach and retrospectively reviewed the effects of GnRHa on final height among girls who experienced earlier puberty, at a single medical center in central Taiwan between 2006 and 2015.

## Materials and methods

### Data source

The data were collected from the records of Pediatric Endocrinology outpatient clinics between January 1^st^, 2006, and December 31^st^, 2015, at the China Medical University Hospital (CMUH), Taichung, Taiwan. CMUH is not only a tertiary university-affiliated medical center but also an institution of medical research and education in central Taiwan.

### Study subjects and variables

We retrospectively reviewed the electronic records of girls with the diagnosis of precocity by the International Classification of Disease, 9^th^ Revision, code of 259.1, at CMUH between January 1^st^, 2006, and December 31^th^, 2015. The collected data contained the date of birth, date of encounter, diagnosis, treatment, height, body weight, puberty stage, hormonal data, skeletal evaluation of the bone age by X-ray, medical prescriptions, maternal height, paternal height, birth weight, and gestational age at birth. In addition, we tracked the final height of the patients using electronic records or telephone contacts.

The inclusion criteria were as follows: (1) Girls; (2) Diagnosis meeting the diagnostic criteria of the ICD-9-CM code of 259.1; (3) A basal serum luteinizing hormone (LH) level≧0.3 IU/L, accepted as activation of the hypothalamic-pituitary-gonadal axis[25]; (4) GnRHa treatment with leuprorelin (3.75 mg/month); (5) Age range from 6 to 12 years old at the first visit. The exclusion criteria were as follows: (1) date of birth after December 31^th^, 2001, to exclude girls who did not attend the final height until December 31^th^, 2015; (2) age younger than 6 years at the first visit because of the difficulty in differentiating girls less than 6 years of age with premature thelarche from those with PP, according to recent literature [25–27]; (3) pathological PP diagnosed by hormonal, abdominal, ultrasound and/or brain image studies; (4) congenital or organic diseases; (5) taking long-term medicine other than GnRHa (leuprorelin, 3.75 mg/month) for more than one month; (6) loss of critical data, for example, bone age.

**Fig 1:**
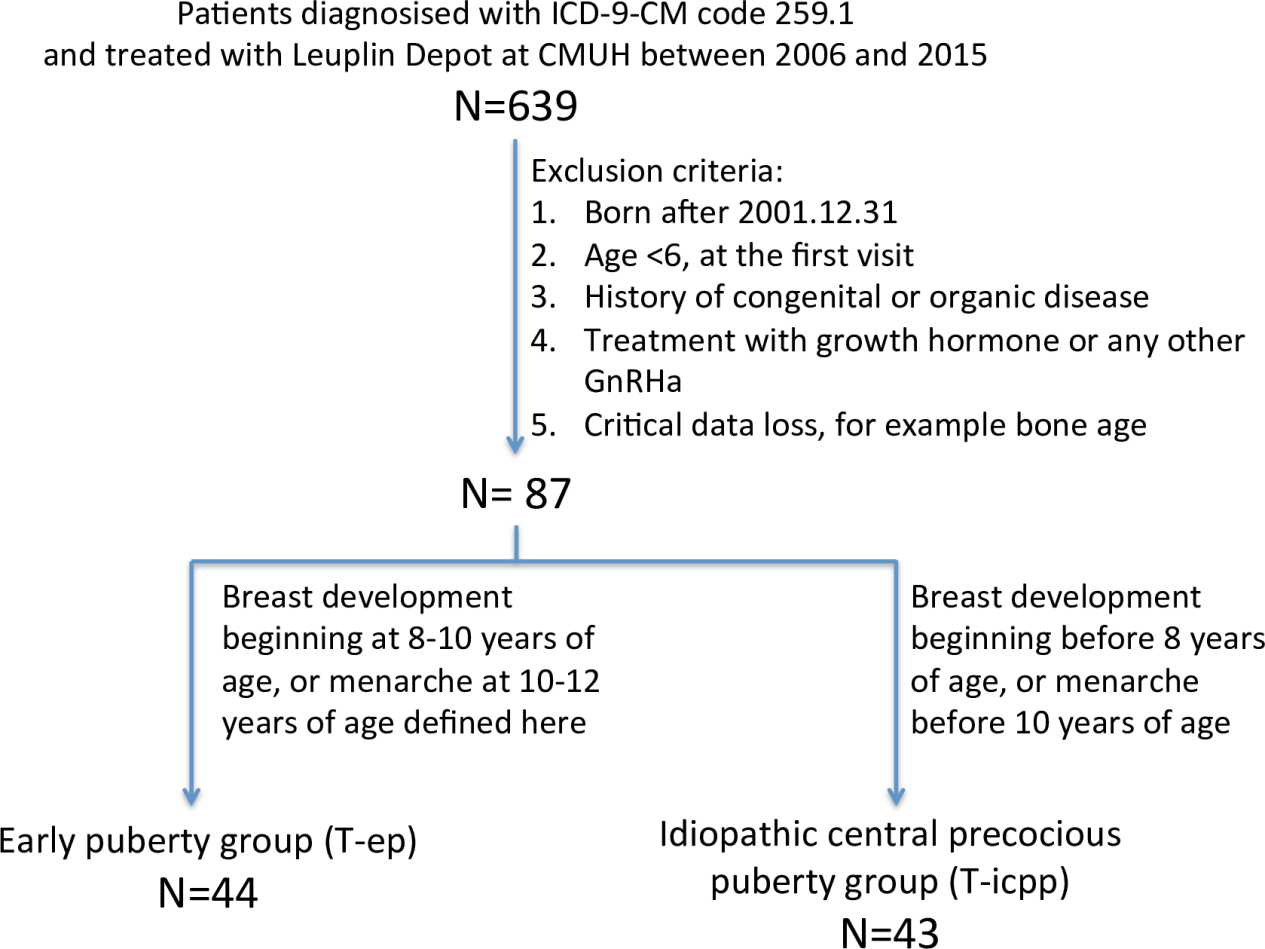
Recruitment flowchart. **Patients diagnosed with PP at CMUH between 2006 and 2015.** Early puberty group (T-ep): Breast development beginning at 8–10 years of age or menarche at 10–12 years of age, as defined here. Idiopathic central precocious puberty group (T-icpp): Breast development beginning before 8 years of age, or menarche before 10 years of age.

Although precocity is defined as the development of secondary sexual characteristics before 8 years of age in girls, we enrolled all of the girls with the diagnosis code of 259.1 who were 6–12 years old at their first visit for potential loss of enrollment in the patient database. In total, 639 patients with a diagnosis of precocity were identified. Of these, 552 patients were excluded. Ultimately, 87 eligible patients were included in this study and divided into two groups according to the age at onset of the first puberty symptoms. The “EP group” (T-ep, T: with GnRHa treatment [leuprorelin]) included 44 patients with breast development beginning at 8–10 years of age or with menarche at 10–12 years of age. The “ICPP group” (T-icpp) included 43 patients with breast development beginning before 8 years of age or with menarche before 10 years of age (Fig 1).

Because this was a retrospective observational study, the allocation of treatment modalities was based on the patient and parent’s choice. During the treatment, patients received more than one skeletal evaluation of their bone age (BA), which were X-ray films of the left hand and wrist that were independently examined by two senior pediatric endocrinologists and a radiologist. Furthermore, the Tanner scale was used for puberty staging, and the height of patients was measured using a wall-mounted stadiometer. Height achieved when the epiphyses were closed and when the growth rate over the last year was <1 cm was considered to represent the final height (FH) [28]. Maternal and paternal heights were recorded at the outpatient follow-ups, and the target height (TH) and standard deviation score (SDS) were determined. The TH was the value of the sum of the patient’s maternal and paternal height divided by two and minus 6.5 cm. The predicted final height (PFH), based on the BA, was calculated according to the Bayley-Pinneau method [29]. The body mass index (BMI) was calculated as kg/m^2^. The height SDS, BMI SDS, and PFH SDS were calculated according to the calendar age. BA/CA was the ratio between bone age and chronological age. The bone maturation ratio (BMR) was calculated as the Δ bone age /Δ chronological age (ΔBA/ΔCA) ratio, compared with the annual change predicted at treatment initiation. PFH and PFH SDS at the first year of treatment and at the final treatment were used to evaluate the short-term effectiveness of the treatment on the heights. The follow-up continued after terminating treatment until the patients reached their FH. The FH and FH SDS were also determined.

### Ethic consideration

This study was approved by the Research Ethics Committee of China Medical University and Hospital (CMUH105-REC1-070). We performed a retrospective analysis using electronic routine clinical and laboratory data at China Medical University Hospital and the data were accessed anonymously. The IRB waived the need for the informed consent forms.

### Statistics

All data were evaluated using the Statistical Package for the Social Sciences 15.0 statistics program (SPSS, Inc., Chicago, IL, USA) in our biostatistics department. Descriptive statistics were used, and the values are presented as the means ± standard deviations or minimum and maximum values. We used a Pearson correlation analysis of the correlation of variables and related factors, such as maternal height, paternal height, birth weight, gestational age at birth and age of maternal menarche. For the two-group analysis, ANCOVA tests were use and a homogeneity test was performed to make the test results accurate, when the mean values were normally distributed in the two groups, using the t-test for comparisons (the independent sample T). A Box plot was used to identify the growth trend during the treatment of height. A p-value < 0.05 was considered significant for all tests.

## Results

### Characteristics of patients with EP or ICPP

The mean age at diagnosis of the total group was 8.76±1.32 years; height, 135.91±9.30 cm; BMI, 18.51±2.76; BA, 123.94±20.81 months; BA/CA, 1.20±0.13; TH, 157.31±3.72 cm; maternal height, 158.26±4.94 cm; paternal height, 169.36±5.63 cm; age of maternal menarche 12.75±1.13 years; birth weight, 3017.15±520.88 g; and gestational age at birth, 39.07±1.92 weeks. The demographic data and anthropometric characteristics of the study groups at the first year of treatment, final treatment, and end of puberty are listed in Table 1. ΔBA/ΔCA at final treatment was significantly lower in the T-ep group than in the T-icpp group (p = 0.017) (Table 1).

**Table 1:** Comparison of the demographic characteristics and anthropometric data of the study groups.

		Total		Early puberty group (T-ep)		Idiopathic central precocious puberty group (T-icpp)	p-value	
		N = 87		n = 44		n = 43		
		MEAN±SD		MEAN±SD		MEAN±SD		
**At the 1**^**st**^ **year of treatment**								
Age (years)		8.93±1.22		9.48 ±0.95	8.36 ±1.23		0.000**	
Estradiol (pg/mL)				49.69±48.912	49.83±27.112			
Luteinizing hormone (mIU/mL)				3.34±3.298	2.25±0.986			
Follicle-stimulating hormone (mIU/mL)				4.36±1.837	4.48±1.779			
Height (cm)		136.92±8.41		139.56 ±6.61	134.14 ±9.25		0.003**	
Height SDS		-1.52±1.00		0.31±0.79	-0.33±1.10		0.003**	
BA (months)		128.01±17.25		131.32 ±11.04	124.28 ±21.85		0.075	
BA/CA		1.19±0.13		1.16 ±0.09	1.23 ±0.15		0.016**	
PFH (cm)		158.55±6.98		159.22 ±6.69	157.82 ±7.29		0.359	
PFH SDS				0.10±0.96	-0.10±1.04		0.359	
BA (months) SDS				0.29 ±0.35	-0.30 ±1.32		0.007**	
**At the time of final treatment**								
Age (years)		11.21±1.32		11.40 ±1.02	11.02 ±1.56		0.181	
Height (cm)		147.44±7.12		148.47 ±5.83	146.39 ±8.18		0.177	
Height SDS		-1.28±1.00		0.14±0.82	-0.15±1.15		0.177	
BA (months)		143.23±14.29		143.07 ±12.94	143.40 ±15.70		0.916	
ΔBA/ΔCA		0.77±0.75		0.57 ±0.39	0.97 ±0.97		0.017**	
PFH		159.63±6.05		160.24 ±6.18	158.99 ±5.92		0.342	
PFH SDS				0.10±1.02	-0.11±0.98		0.342	
Duration of treatment (months)		27.39±14.54		23.05 ±10.43	31.84 ±16.78		0.005**	
**At the end of puberty**								
Final height (cm)		159.95±5.44		160.96 ±5.35		158.98 ±5.45	0.206	
Final height SDS				0.19±0.98		-0.18±1.00	0.206	
Target height (cm)		157.31±3.72		156.83 ±3.94		157.79 ±3.48	0.341	
Target height SDS				-0.13 ±1.06		0.13 ±0.94	0.341	

SDS: standard deviation score, BA: bone age, CA: chronological age, PFH: predicted final height

**: p-value < 0.05

The change in the height SDS from the start of treatment to the final treatment and the difference between the FH SDS and the TH SDS are presented in Table 2.

**Table 2:** Change in the predicted final height SDS between the initial and the final treatment and the difference between the final height SDS and the target height SDS.

Variables	Early puberty group (T-ep)	P-value	Idiopathic central precocious puberty group (T-icpp)	P-value
	MEAN±SE		MEAN±SE	
Gap in the PFH SDS between the 1^st^ and final treatments	0.004±0.11^#1^	0.990	-0.04±0.14 ^#3^	0.434
Gap between the FH SDS and the TH SDS	0.35±0.21 ^#2^	0.045*	-0.24±0.16^#4^	0.145

PFH = predicted final height, FH = final height, TH = target height, SDS = standard deviation scores.

#1 notes N = 44

#2 notes N = 23

#3 notes N = 40

#4 notes N = 25

*: p-value < 0.05

**Fig 2:**
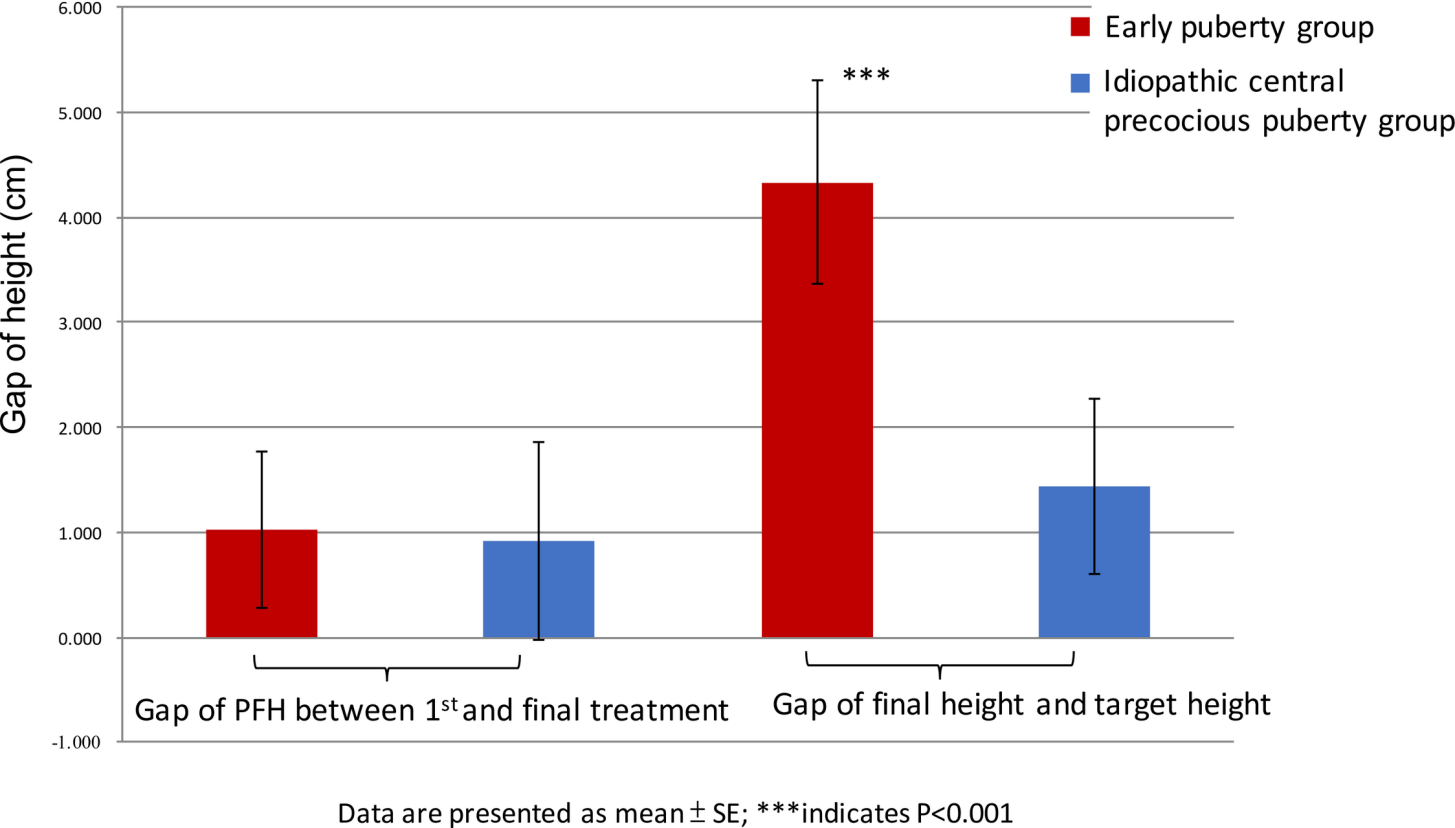
Change in predicted final height between the initial and final treatments and difference between final height and target height.

The change in height from the start of treatment to the final treatment and the difference between the FH and the TH is shown in Fig 2. We found that only the early puberty group had a significant improvement in final height, compared with the target height.

**Fig 3:**
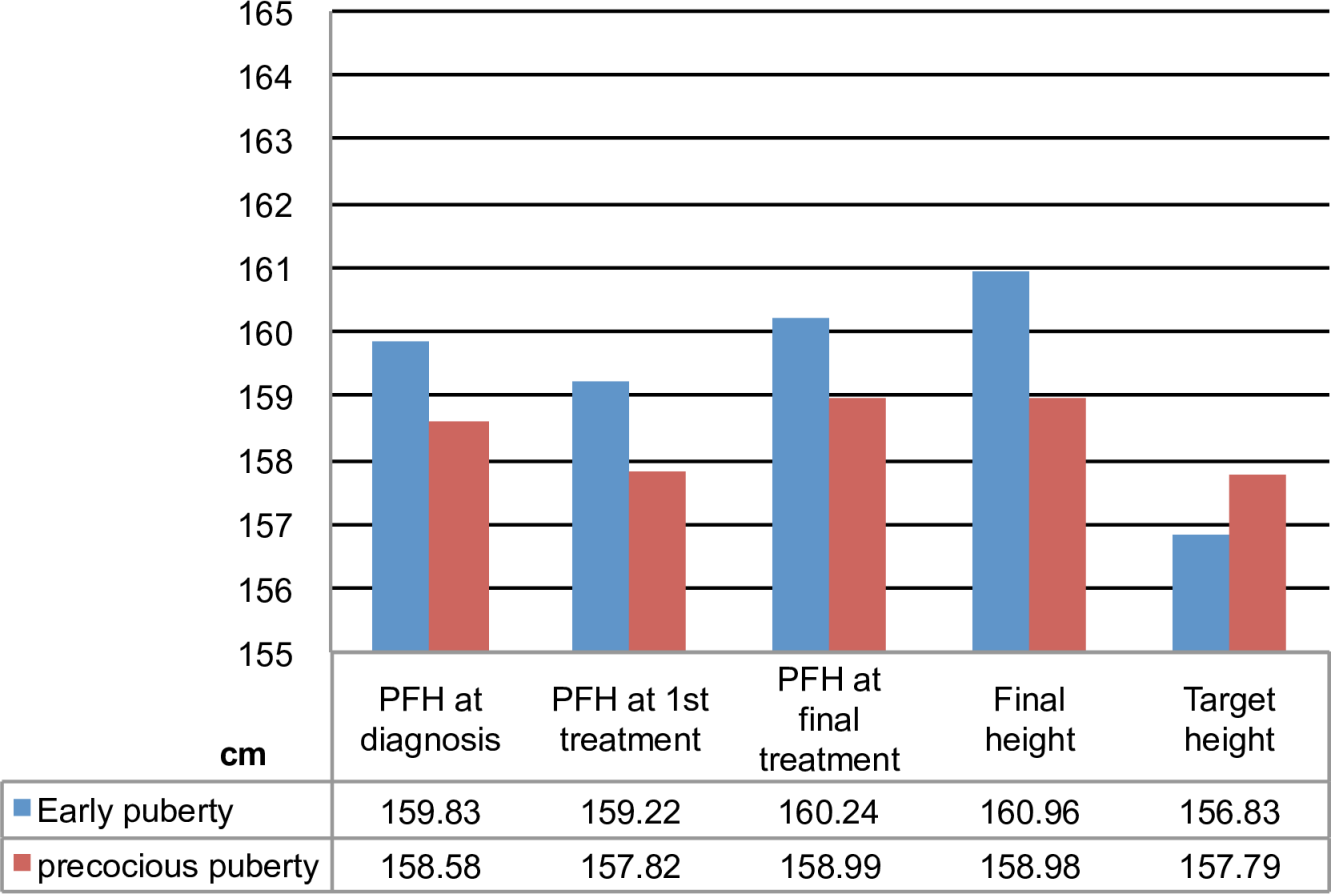
Summary of the change in predicted final height at different stages, the final height and the target height.

In addition, the summary of the change in final height predicted by the bone age at different stages, the final height and the target height is presented in Fig 3.

We have also summarized the information on the baseline data and the differences in outcome among other studies and our study in Table 3.

**Table 3:** Summary of studies on GnRHa treatment of girls with advanced puberty.

				Baseline Characteristics			Outcomes	
Authors (Year)	Treatment	Duration of Therapy	Subjects (n)	Age (years)	Height (cm)	Bone age	PFH (cm)	FH (cm)
Mul (2005)	Triptorelin 3.75 mg/28 days, IM	3 years	12	9.6±0.9	133.8±8.7	10.7±1.1 years	149.8±5.6	155.0±5.6
Pucarelli (2003)	Triptorelin 100 mg/kg/21 days, IM	2–4 years	18	9.9±0.9	-	10.7±0.8 years	149.6±4.0	156.6±5.7
Bouvattier (1999)	Triptorelin 3.75 mg/28 days, IM	2 years	20	9.3±0.5	135.2±4.3	10.9±0.5 years	154.1±3.9	157.6±4.0
Our study (2017)	Leuprorelin 3.75 mg/28 days, IM	23.05±10.43 months	87	9.36 ±1.0	138.83±7.06	130.43±12.49 months	159.83±7.19	160.96±5.35

IM = intra-muscularly, PFH = predicted final height. Data are presented as the mean ± standard deviation unless otherwise stated.

## Discussion

Earlier puberty is associated with reduced adult height, metabolic syndrome, and psychological problems. In the clinical practice, many girls with EP are referred to a pediatric endocrinologist. However, previous literature regarding the effectiveness of GnRHa treatment in children with EP is lacking. Our retrospective study showed that GnRHa treatment was beneficial for girls with earlier puberty, which was especially noted in the T-ep group, by reducing BMR (= ΔBA/ΔCA) to circumvent the negative effects on height and increase the PFH after GnRHa treatment. This is the first study to evaluate the effectiveness of GnRHa treatment in patients with earlier puberty in the Han Chinese population residing in Taiwan.

Our results showed that in the T-ep group, the mean age at diagnosis, mean bone age at baseline, and duration of therapy were consistent with a number of studies, such as investigations by Mul et al. [30], Pucarelli et al. [31] and Bouvattier et al. [20], as listed in Table 3. While height (138.83±7.06 cm) at diagnosis, PFH (159.83±7.19 cm) at diagnosis, and FH (160.96±5.35 cm) (Table 1) in this study were higher than those in earlier studies. The final height gain (approximately 22 cm) from the baseline to the commencement of treatment appears to be similar in our study and in an additional two studies (Mul et al, Bouvattier et al) and may demonstrate the comparable treatment effects between different races and eras. Overall, our study provides valuable information and contributes to the existing knowledge regarding health care in PP patients. The increased height may stem from a number of reasons. First, different periods and environments: the average height of boys and girls at 18 years of age in Taiwan, in the Netherlands, in Italy, and in France, are higher than in the past. This is a secular trend, according to recent research [32]. Second, Taiwanese children ingest adequate food with improved macro- and micronutrient supplements and have easier medical access than in the past, all of which contributed to the adverse effects on growth. Furthermore, this generation has enjoyed economic prosperity, and height is affected by fetal and early childhood nutrition [33], health and economic productivity [34–36].

In this study, we found that the value of BMR (ΔBA/ΔCA) at final treatment decreased in both groups. Additionally, the deceleration of ΔBA/ΔCA after final treatment was significantly greater in the T-ep group than in the T-icpp group (0.57 ±0.39 vs. 0.97 ±0.97, p = 0.017) (Table 1). This finding indicated that the efficacy of GnRHa to suppress BMR was more greater in the T-ep group than in the T-icpp group. However, there was no significant difference between the T-icpp and T-ep groups with respect to the values of PFH during treatment and with respect to the FH, despite improved bone age deceleration in the T-ep group. Furthermore, both groups tended to reach a higher final height than that predicted after final treatment (Fig 3). Compared with the T-icpp, a greater height at the time of diagnosis and a slower BA progression with treatment in the T-ep did not yield a greater gain in final height. In addition, it appeared that the FH or height gain during treatment in these age groups could not be judged only by the deceleration of bone age advancement. However, we did observe that both treated groups reached the TH, with the T-ep group even achieving a final height of 4.13 cm greater than expected (mean TH: 156.83±3.94 cm), and the bone age at the last final treatment could be used to predict the adult final height (Fig 3).

There are several limitations to our study. The main limitation was its retrospective design. In addition, the lack of an untreated age- and BMI-matched control group has raised unanswered questions regarding the effectiveness of treatment in these age groups and the accuracy of the height prediction model. Furthermore, we only included girls, and different factors, which are more likely to be pathological in origin, may be involved in boys with CPP and EP. We also did not investigate other confounding factors, such as subject lifestyle, including extent of physical activity and dietary habits. Additionally, a potential risk of selection bias may exist because of the limited sample size from a single center. Therefore, it is difficult to determine the factors playing a major role in our findings, although they might be the effect of genetic and environmental factors. Finally, the subject population observed at the final adult height was relatively small.

## Conclusion

We found that GnRHa treatment contributed to BMR reduction to improve the PFH for girls with earlier puberty, especially in the T-ep group. Our results should provide valuable information for pediatric endocrinologists, parents and the government concerning the health care of children with earlier puberty. Further research is warranted to examine the efficacy and safety of GnRHa in children with earlier puberty.
